# Effect of the COVID-19 outbreak on emergency transport of children by an emergency medical service system: a population-based, ORION registry study

**DOI:** 10.1186/s12873-022-00765-8

**Published:** 2022-12-20

**Authors:** Koshi Ota, Daisuke Nishioka, Yusuke Katayama, Tetsuhisa Kitamura, Jun Masui, Kanna Ota, Masahiko Nitta, Tetsuya Matsuoka, Akira Takasu

**Affiliations:** 1 Department of Emergency Medicine, Osaka Medical and Pharmaceutical University, Takatsuki, Japan; 2 The Working Group to Analyze the Emergency Medical Care System in Osaka Prefecture, Osaka, Japan; 3 Research and Development Center, Osaka Medical and Pharmaceutical University, Takatsuki, Japan; 4 Osaka Prefectural Government, Osaka, Japan

**Keywords:** COVID-19, Children, Difficult-to-transfer cases, Pandemic

## Abstract

**Background:**

Coronavirus disease 2019 (COVID-19), caused by Severe Acute Respiratory Syndrome Coronavirus 2, has spread rapidly around the world.

**Objective:**

To assess the effect of the COVID-19 pandemic on the emergency medical service (EMS) and hospital admission course for children transported by ambulance.

**Methods:**

This study was a retrospective, descriptive study with a study period from January 1, 2018 to December 31, 2020 using the Osaka Emergency Information Research Intelligent Operation Network (ORION) system. All children who were transported by ambulance in Osaka Prefecture were included. The main outcome of this study was the rate of difficult-to-transfer cases, which was calculated by univariate and multivariate Poisson regression analyses.

**Results:**

Over the 3 years between January 1, 2018 and December 31 2020, 1,436,212 patients were transported to hospitals by ambulances in Osaka Prefecture, with children accounting for 102,473 (37,064, 39,590, and 25,819, in 2018, 2019, and 2020, respectively). Poisson regression analysis showed that children were negatively associated with difficult-to-transfer cases (risk ratio (RR) 0.35, 95% CI 0.33 to 0.37). With reference to 2018, 2020 was not significantly associated with difficult-to-transfer cases in children (RR 1.14, 95% CI 0.99 to 1.32, P = 0.075), but was significantly related (RR 1.24, 95% CI 1.21 to 1.27, P < 0.001) to difficult-to-transfer cases in the general population.

**Conclusion:**

Children were consistently associated with a reduced RR for difficult-to-transfer cases, even in the COVID-19 pandemic in 2020.

**Supplementary Information:**

The online version contains supplementary material available at 10.1186/s12873-022-00765-8.

## Introduction

Coronavirus disease 2019 (COVID-19), causedby Severe Acute Respiratory Syndrome Coronavirus 2 (SARS-CoV-2), was identified in China in December 2019, and the COVID-19 outbreak spread rapidly around the world [1]. On March 11, 2020, the World Health Organization (WHO) declared COVID-19 a pandemic.

Children with COVID-19 tend to have a milder clinical course than adults, and the clinical manifestations in children are heterogeneous, with a wide spectrum of clinical features [2–5]. The WHO recommends testing all suspected cases, but children with COVID-19 may not meet all the criteria of the suspected case definition. [6, 7]

A previous study in Osaka city showed a similar proportion of children transported by the emergency medical service (EMS) system to that seen in other countries, about 4% to 13% [8–10]. Our previous study showed about a 10% reduction in EMS service use in the overall population, including elderly patients, in 2020, possibly due to the COVID-19 pandemic [11]. However, EMS service studies related to the COVID-19 pandemic and children have been scarce.

Presently, to the best of our knowledge, no clinical studies of the effect of the COVID-19 outbreak and transport of children by the EMS system have been published.

This study aimed to assess the effect of the COVID-19 pandemic on the transport by ambulance of children by the EMS system in Osaka Prefecture.

## Methods

### The emergency medical service (EMS) system in Japan

When emergency patients call for EMS at the scene, on-scene EMS personnel assess the patient’s condition and then transport the patient to a hospital that can accept and treat the patient [12]. Only after obtaining permission from the selected hospital via a phone call, ambulances can transport the patient to the hospital [12]. All expenses are covered by local governments, and there is no charge to the patient for care and transportation. Recently, especially COVID-19 pandemic started, the number of cases of emergency patients’ transportation to a hospital by EMS has been increasing and exceeding the hospital capacity. Therefore, it has been increasing cases of refusal to provide emergency transportation.

### Study design and setting

**Fig. 1:**
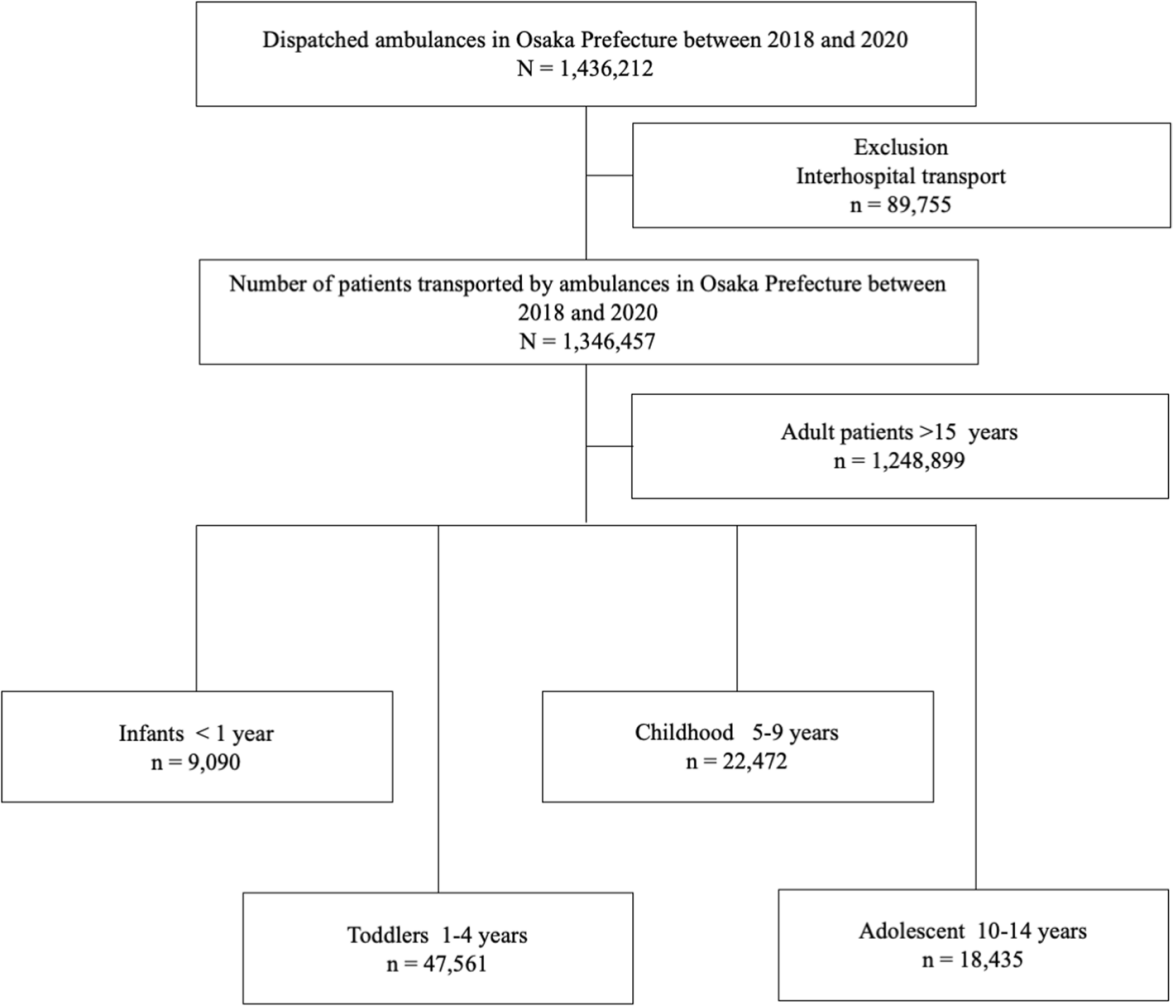
Patient flow in this study. All pediatric patients aged less than 15 years were included and then divided into four groups by age

This was a retrospective, descriptive study with a study period from January 1, 2018 to December 31, 2020 using the Osaka Emergency Information Research Intelligent Operation Network (ORION) system [13]. Osaka Prefecture is the largest metropolitan community in western Japan, with a population of about 8.8 million and a total area of 1905 km [2]. The Osaka Prefecture Government has developed and introduced an information system for emergency patients (the ORION system) that uses a smartphone application for hospital selection by on-scene emergency medical service personnel and has been collecting all ambulance records. Medical institutions have obtained information on the diagnoses and outcomes of patients transported to medical institutions, and the ORION system has merged these data with ambulance records, including smartphone application data, since January 2015. To assess the effect of the COVID-19 pandemic on the EMS system, this study focused on children transported by ambulance in Osaka Prefecture (Fig. 1). The children were divided into four age groups: infant (0 years); toddler (1–4 years); childhood (5–9 years); and adolescent (10–14 years). The presumptive diagnosis and the final diagnosis if the patients were admitted, with the International Classification of Diseases, 10th Revision (ICD-10), were used [14]. Data were collected using ICD-10 codes: Infectious and parasitic diseases, A00 to B99; Neoplasms, C00 to D48; Endocrine, nutritional and metabolic diseases, E00 to E90; Mental and behavioral disorders, F00 to F99; Diseases of the nervous system, G00 to G99; Diseases of the eye and the ear, H00 to H95; Diseases of the nervous system, I00 to I99; Diseases of the respiratory system, J00 to J99; Diseases of the digestive system, K00 to K93; Diseases of the skin and subcutaneous tissue, L00 to L99; Diseases of the musculoskeletal system and connective tissue, M00 to M99; Diseases of the genitourinary system, N00 to N99; Congenital diseases, Q00 to Q99; Symptoms, signs and abnormal clinical and laboratory findings, R00 to R99; Injury and poisoning, S00 to T98. ICD-10 codes O00 to O99 and P00 to P96 were excluded because these codes were defined as pregnancy-related cases (pregnant patients). Data for ‘COVID-19’ were collected using ICD-10 code U07.1, and data for ‘COVID-19 suspected’ (if the virus was not identified) were collected using ICD-10 code U07.2.

This study excluded patients who were not transported to a hospital. The ambulance records in Osaka Prefecture are considered administrative records, and the need to obtain informed consent from the participants was waived because the data were anonymous. This study was approved by the Ethics Committee of Osaka Medical and Pharmaceutical University (Takatsuki City, Japan) (reference number 2021–006). The Strengthening the Reporting of Observational studies in Epidemiology (STROBE) guidelines were used to design and report the results of this study [15].

### Data collection and quality control

The ORION system uses a smartphone app for hospital selection by on‐scene EMS personnel and it has been accumulating all ambulance records. Data were uniformly collected using specific data collection forms and included the reason for the ambulance call, location of the accident, the time of day and the day of the week, and tools used, in addition to age, sex, and ICD-10 code. The detailed situation and patient information were recorded in text form. These data were completed by EMS personnel and then transferred to the information center in the Osaka Municipal Fire Department (OMFD). Medical institutions have registered information on the diagnosis and outcome of emergency patients transported to medical institutions, and the ORION system has merged these data with the respective ambulance records and smartphone app data. To assure the quality of the data, if the data were incomplete, the data sheet was supposed to be returned to the relevant EMS personnel to correct it.

### Outcomes

The primary outcome of this study was the rate of difficulty obtaining hospital acceptance for transfer of a patient (difficult-to-transfer cases). According to the guidelines of the Fire and Disaster Management Agency of the Ministry of Internal Affairs and Communications, “difficult-to-transfer cases” were defined as those in which the time interval from arrival at the scene to departure from the scene was longer than 30 min, and those in which ambulance crews needed to make four or more phone calls to hospitals before obtaining hospital acceptance. In addition, disposition at the emergency department (ED), such as emergency admission, discharged to home, transfer to other hospital, and death at the ED, was also collected.

### Data analysis

The number of patients transported by ambulance due to any cause except interhospital transport was calculated for the year from January 1 to December 31, 2020. Patient demographics were compared among the three years using the χ^2^ test for categorical variables and the Kruskal–Wallis test for continuous variables. For comparison purposes, the numbers of patients transported by ambulance for the same reasons per year from January 1 to December 31, 2018 and January 1 to December 31, 2019 were also collected. In addition, Poisson regression analysis with a robust standard error estimator was used to calculate the rate of difficulty of hospital acceptance of patients, and then the risk ratio (RR) for difficult-to-transfer cases and the 95% confidence interval (CI) were calculated with 2018 as the reference. Then, the adjusted RR and 95% CI of difficult-to-transfer cases by month, time of transportation, day of week, COVID-19 suspected during transportation, and children were estimated using multivariate analyses.

All data were analyzed using SPSS version 25.0 software (IBM Corp., Armonk, NY, USA) or STATA (version 16.1; Stata Corp., College Station, TX, USA). All tests were two-tailed, and *p*-values < 0.05 were considered significant.

## Results

### Baseline characteristics

For 3 years between January 1, 2018 and December31, 2020, 1,436,212 patients were transported to hospitals by ambulances in Osaka Prefecture. Of them, 1,346,457 were enrolled in this study. Excluded were 89,755 patients who were transferred to a different hospital. By year, 462,773 patients in 2018, 468,697 patients in 2019, and 414,987 patients in 2020 were transported to hospitals by ambulances (Table 1), with the total number of children (0–14 years old) being 97,558 (35,314, 37,547, and 24,697, in 2018, 2019, and 2020, respectively, *P* < 0.001). In addition, the total number of infants was 9,090 (3,290, 3,375, and 2,425, in 2018, 2019, and 2020, respectively). Table 1 shows other baseline characteristics of the pediatric patients transported to hospitals by ambulances in Osaka Prefecture. Figure 1 displays the flow diagram of this study.

**Table 1:** Demographic characteristics of transported patients

Year	2018	2019	2020	Total	*P* value
Number of Patients	462,773	468,697	414,987	1,346,457	< 0.001
Age, median (IQR)	69.0(38)	70.0(38)	71.0(35)		< 0.001
Sex (male), %	234,542 (50.7)	236,661 (50.5)	210,334 (50.7)	681,537 (50.6)	0.111
Age, median (IQR)	67.0 (38)	67.0 (38)	69.0 (34)		< 0.001
Children, %	35,314 (7.6)	37,547 (8.0)	24,697 (6.0)	97,558 (7.3)	< 0.001
Age, median (IQR)	3.0 (7)	3.0 (7)	4.0 (7)		< 0.001
Adult, %	166,402 (36.0)	164,722 (35.1)	143,740 (34.6)	474,864 (35.3)	< 0.001
Age, median (IQR)	43.0 (26)	44.0 (26)	45.0 (26)		< 0.001
Elderly patients, %	261,057 (56.4)	266,428 (56.8)	246,550 (59.4)	774,035 (57.5)	< 0.001
Age, median (IQR)	80.0 (12)	80.0 (12)	80.0 (12)		< 0.001
Children age category					< 0.001
Infant, %	3,661 (9.9)	3,790 (9.6)	2,641 (10.2)	10,092 (9.9)	
Toddler, %	17,964 (48.5)	19,803 (50.0)	11,873 (46.0)	49,640 (48.4)	
Childhood, %	8,508 (23.0)	8,879 (22.4)	6,146 (23.8)	23,533 (23.0)	
Adolescent, %	6,931 (18.7)	7,118 (18.0)	5,159 (20.0)	19,208 (18.7)	
Reason for transportation (Children)					
Infectious and parasitic diseases, %	2,009 (5.8)	2,602 (7.0)	1,065 (4.4)	5,676 (5.9)	< 0.001
Neoplasms, %	59 (0.2)	56 (0.2)	57 (0.2)	172 (0.2)	0.052
Endocrine, nutritional, and metabolic diseases, %	253 (0.7)	251 (0.7)	167 (0.7)	671 (0.7)	0.713
Mental and behavioral disorders, %	430 (1.3)	389 (1.1)	267 (1.1)	1,086 (1.1)	0.056
Diseases of the nervous system, %	912 (2.7)	1,018 (2.7)	892 (3.7)	2,822 (2.9)	< 0.001
Diseases of the eye and the ear, %	152 (0.4)	164 (0.4)	87 (0.4)	403 (0.4)	0.224
Diseases of the circulatory system, %	200 (0.6)	238 (0.6)	190 (0.7)	662 (0.6)	0.199
Diseases of the respiratory system, %	4,224 (12.3)	4,798 (12.9)	2,252 (9.3)	11,274 (11.8)	< 0.001
Diseases of the digestive system, %	858 (2.5)	921 (2.5)	712 (2.9)	2,491 (2.6)	0.001
Diseases of the skin and subcutaneous tissue, %	349 (1.0)	380 (1.0)	229 (0.9)	958 (1.0)	0.57
Diseases of the musculoskeletal system and connective tissue, %	404 (1.2)	458 (1.2)	350 (1.4)	1,212 (1.3)	0.011
Diseases of the genitourinary system, %	130 (0.4)	134 (0.4)	145 (0.6)	409 (0.4)	< 0.001
Congenital diseases, %	23 (0.1)	32 (0.1)	23 (0.1)	78 (0.1)	0.441
Symptoms, signs, and abnormal clinical and laboratory findings, %	9,925(28.8)	11,171 (30.1)	6,276 (25.8)	27,372 (28.5)	< 0.001
Injury and poisoning, %	14,542 (42.2)	14,536 (39.2)	11,676 (47.9)	40,754 (42.5)	< 0.001

Children, 0–14 years; Adults, 15–64 years; Elderly patients, ≥ 65 years

Infant, 0 years; Toddler, 1–4 years; Childhood, 5–9 years; Adolescent, 10–14 years

Determined by the χ^2^ test for categorical variables and Kruskal–Wallis test for continuous variables

*Abbreviation*: *IQR* interquartile range

It was difficult to obtain hospital acceptance for transfer of a total of 1,147 pediatric patients (397, 433, and 317, in 2018, 2019, and 2020, respectively, *P* = 0.179) (Table 2).

**Table 2:** Difficult-to-transfer cases for children and hospital course

Year	2018		2019		2020		Total		*P* value
All	35,314		37,547		24,697		97,558		
Not difficult-to-transfer cases, %	34,917	(98.9)	37,114	(98.9)	24,380	(98.7)	96,411	(98.8)	0.179
At the scene ≥ 30 min	583	(1.7)	635	(1.7)	358	(1.4)	1576	(1.6)	
≥ 4 phone calls	704	(2.0)	651	(1.7)	514	(2.1)	1869	(1.9)	
Difficult-to-transfer cases, %	397	(1.1)	433	(1.2)	317	(1.3)	1147	(1.2)	
Disposition at the ED									< 0.001
Admission, %	5,708	(16.2)	6,186	(16.5)	4,467	(18.1)	16,361	(16.8)	
Home, %	29,385	(83.2)	31,143	(83.0)	20,063	(81.2)	80,591	(82.6)	
Transfer, %	178	(0.5)	172	(0.5)	133	(0.5)	483	(0.5)	
Death, %	42	(0.1)	45	(0.1)	34	(0.1)	121	(0.1)	

Determined by the χ^2^ test for categorical variables

Table 2 also shows the disposition at the ED.

The number of children who were suspected to have COVID-19 during transportation was 43 (4 infants, 20 toddlers, 8 childhoods, and 11 adolescents). Of these 43, only one adolescent was a difficult-to-transfer case.

### Outcomes and adjusted analyses

The RR of difficult-to-transfer cases for pediatric patients in 2020 was not significant with reference to 2018 (1.16, 95%CI 0.74 to 1.82) (Table 3). Table 5 shows the same analysis as Table 4 for children only, and there was no significant association with difficult-to-transfer cases in 2020 (RR 1.14, 95% CI 0.98 to 1.32, *P* = 0.081). Different from the general population, children were more accepted by hospitals in January and December than in June. With reference to 9 to 10 am, 3 pm to 2 am was significantly associated with difficult-to-transfer cases for children (Table 5). Similar to the general population, with reference to Friday, Saturday and Sunday had significantly higher RRs for difficult-to-transfer cases (RR 1.37, 95% CI 1.11 to 1.70 and RR 1.37, 95% CI 1.11 to 1.70, respectively). There was no significant association between pediatric patients suspected to have COVID-19 and difficult-to-transfer cases (RR 1.72, 95% CI 0.25 to 12.08, *P* = 0.584).

**Table 3:** Univariate Poisson regression analysis for difficult-to-transfer cases and disposition at the emergency department

	Risk Ratio	95% confidence interval			*P* value
**Difficult-to-transfer cases**					
Children, < 15 years					
2018	Reference				
2019	1.03	0.90	−	1.17	0.712
2020	1.14	0.99	−	1.32	0.077
Adults, ≥ 15 years					
2018	Reference				
2019	0.92	0.90	−	0.94	< 0.001
2020	1.25	1.22	−	1.28	< 0.001
**Disposition at the Emergency Department**					
**Admission**					
Children, < 15 years					
2018	Reference				
2019	1.02	0.99	−	1.05	0.255
2020	1.12	1.08	−	1.16	< 0.001
Adults, ≥ 15 years					
2018	Reference				
2019	1.02	1.01	−	1.02	< 0.001
2020	1.08	1.07	−	1.08	< 0.001
**Home**					
Children, < 15 years					
2018	Reference				
2019	1.00	0.99	−	1.00	0.337
2020	0.98	0.97	−	0.98	< 0.001
Adults, ≥ 15 years					
2018	Reference				
2019	0.99	0.98	−	0.99	< 0.001
2020	0.94	0.94	−	0.95	< 0.001
**Death**					
Children, < 15 years					
2018	Reference				
2019	1.01	0.66	−	1.53	0.971
2020	1.16	0.74	−	1.82	0.526
Adult, ≥ 15 years					
2018	Reference				
2019	1.00	0.96	−	1.04	0.945
2020	1.22	1.17	−	1.27	< 0.001

**Table 4:** Multivariate Poisson regression analysis of difficult-to-transfer cases in all patients

	Risk Ratio	95% confidence interval			*P* value
**Year**					
2018	Reference				
2019	0.93	0.90	−	0.95	< 0.001
2020	1.24	1.21	−	1.27	< 0.001
**Month**					
June	Reference				
January	1.93	1.84	−	2.02	< 0.001
February	1.85	1.76	−	1.94	< 0.001
March	1.49	1.41	−	1.57	< 0.001
April	1.59	1.51	−	1.67	< 0.001
May	1.39	1.32	−	1.47	< 0.001
July	1.14	1.08	−	1.20	< 0.001
August	1.41	1.34	−	1.49	< 0.001
September	1.20	1.14	−	1.27	< 0.001
October	1.12	1.06	−	1.18	< 0.001
November	1.28	1.21	−	1.35	< 0.001
December	1.52	1.45	−	1.60	< 0.001
**Time of transportation**					
9 am to 10 am	Reference				
0 am to 1 am	6.44	5.91	−	7.02	< 0.001
1 am to 2 am	7.42	6.80	−	8.08	< 0.001
2 am to 3 am	7.95	7.29	−	8.67	< 0.001
3 am to 4 am	7.66	7.02	−	8.37	< 0.001
4 am to 5 am	7.61	6.96	−	8.31	< 0.001
5 am to 6 am	6.80	6.21	−	7.44	< 0.001
6 am to 7 am	5.49	5.01	−	6.00	< 0.001
7 am to 8 am	3.98	3.63	−	4.36	< 0.001
8 am to 9 am	2.16	1.97	−	2.38	< 0.001
10 am to 11 am	1.25	1.13	−	1.38	< 0.001
11 am to 12 pm	1.58	1.44	−	1.75	< 0.001
12 pm to 1 pm	1.92	1.75	−	2.11	< 0.001
1 pm to 2 pm	2.18	1.98	−	2.39	< 0.001
2 pm to 3 pm	2.21	2.01	−	2.42	< 0.001
3 pm to 4 pm	2.41	2.19	−	2.64	< 0.001
4 pm to 5 pm	2.41	2.19	−	2.64	< 0.001
5 pm to 6 pm	3.08	2.82	−	3.37	< 0.001
6 pm to 7 pm	3.85	3.54	−	4.20	< 0.001
7 pm to 8 pm	4.48	4.12	−	4.88	< 0.001
8 pm to 9 pm	4.81	4.42	−	5.23	< 0.001
9 pm to 10 pm	4.86	4.46	−	5.29	< 0.001
10 pm to 11 pm	5.25	4.82	−	5.72	< 0.001
11 pm to 0 am	5.76	5.29	−	6.28	< 0.001
**Day of week**					
Friday	Reference				
Monday	1.11	1.07	−	1.15	< 0.001
Tuesday	1.08	1.04	−	1.12	< 0.001
Wednesday	1.09	1.05	−	1.13	< 0.001
Thursday	1.06	1.02	−	1.10	0.004
Saturday	1.26	1.21	−	1.30	< 0.001
Sunday	1.38	1.34	−	1.43	< 0.001
Adults, ≥ 15 years	Reference				
Children	0.35	0.33	−	0.37	< 0.001
Suspected COVID-19	2.50	2.27	−	2.75	< 0.001

**Table 5:** Multivariate Poisson regression analysis of difficult-to-transfer cases in children

	Risk Ratio	95% confidence interval			*P* value
**Year**					
2018	Reference				
2019	1.03	0.90	−	1.18	0.704
2020	1.14	0.98	−	1.32	0.081
**Month**					
June	Reference				
January	0.75	0.57	−	0.99	0.041
February	0.82	0.62	−	1.09	0.18
March	1.16	0.89	−	1.50	0.28
April	0.90	0.68	−	1.19	0.446
May	0.92	0.70	−	1.21	0.549
July	0.83	0.63	−	1.09	0.18
August	0.90	0.69	−	1.19	0.466
September	1.02	0.78	−	1.34	0.888
October	1.01	0.77	−	1.33	0.931
November	1.03	0.79	−	1.36	0.806
December	0.72	0.54	−	0.97	0.03
**Time of transportation**					
9 am to 10 am	Reference				
0 am to 1 am	2.26	1.23	−	4.13	0.008
1 am to 2 am	2.15	1.15	−	4.03	0.017
2 am to 3 am	1.94	0.99	−	3.82	0.053
3 am to 4 am	1.56	0.74	−	3.27	0.238
4 am to 5 am	0.82	0.32	−	2.12	0.689
5 am to 6 am	1.07	0.44	−	2.63	0.874
6 am to 7 am	0.72	0.26	−	1.98	0.528
7 am to 8 am	0.67	0.26	−	1.73	0.414
8 am to 9 am	1.30	0.66	−	2.57	0.453
10 am to 11 am	1.31	0.68	−	2.51	0.423
11 am to 12 pm	1.35	0.72	−	2.52	0.346
12 pm to 1 pm	2.06	1.16	−	3.67	0.013
1 pm to 2 pm	1.54	0.86	−	2.77	0.147
2 pm to 3 pm	1.31	0.72	−	2.37	0.376
3 pm to 4 pm	2.16	1.24	−	3.79	0.007
4 pm to 5 pm	2.06	1.18	−	3.60	0.011
5 pm to 6 pm	2.76	1.61	−	4.74	< 0.001
6 pm to 7 pm	3.16	1.85	−	5.39	< 0.001
7 pm to 8 pm	3.17	1.85	−	5.41	< 0.001
8 pm to 9 pm	2.54	1.48	−	4.38	0.001
9 pm to 10 pm	3.05	1.78	−	5.24	< 0.001
10 pm to 11 pm	3.71	2.16	−	6.38	< 0.001
11 pm to 0 am	3.08	1.76	−	5.40	< 0.001
**Day of week**					
Friday	Reference				
Monday	1.18	0.94	−	1.48	0.156
Tuesday	0.98	0.77	−	1.25	0.895
Wednesday	0.95	0.75	−	1.21	0.687
Thursday	1.18	0.94	−	1.49	0.151
Saturday	1.37	1.11	−	1.70	0.004
Sunday	1.37	1.11	−	1.70	0.003
Suspected COVID-19	1.72	0.25	−	12.08	0.584

In children, female sex was associated with difficult-to-transfer cases (RR 1.20, 95% CI 1.07 to 1.35, *P* = 0.002) (Table 6). With reference to injury, poisoning and certain other consequences of external causes, diseases of the genitourinary system and diseases of the musculoskeletal system and connective tissue were positively associated with difficult-to-transfer cases (RR 1.66, 95% CI 1.01 to 2.75, *P* = 0.047 and RR 1.53, 95% CI 1.13 to 2.08, *P* = 0.006, respectively). Other diseases were inversely or not significantly associated with difficult-to-transfer cases (Table 6).

**Table 6:** Multivariate Poisson regression analysis of difficult-to-transfer cases in children

Difficult-to-transfer cases	Risk Ratio	95% confidence interval			*P* value
Year					
2018	Reference				
2019	1.07	0.93	−	1.22	0.93
2020	1.01	0.87	−	1.17	0.17
Injury and poisoning	Reference				
Infectious and parasitic diseases	0.10	0.06	−	0.17	< 0.001
Neoplasms	0.51	0.13	−	2.03	0.338
Endocrine, nutritional, and metabolic diseases	0.33	0.14	−	0.79	0.013
Mental and behavioral disorders	0.60	0.36	−	1.00	0.049
Diseases of the nervous system	0.16	0.08	−	0.29	< 0.001
Diseases of the eye and the ear	0.88	0.44	−	1.75	0.708
Diseases of the circulatory system	0.16	0.04	−	0.63	0.009
Diseases of the respiratory system	0.08	0.05	−	0.13	< 0.001
Diseases of the digestive system	0.25	0.15	−	0.42	< 0.001
Diseases of the skin and subcutaneous tissue	0.05	0.01	−	0.33	0.002
Diseases of the musculoskeletal system and connective tissue	1.53	1.13	−	2.08	0.006
Diseases of the genitourinary system	1.66	1.01	−	2.75	0.047
Congenital diseases	0.57	0.08	−	3.97	0.569
Symptoms, signs, and abnormal clinical and laboratory findings	0.12	0.09	−	0.15	< 0.001
Male	Reference				
Female	1.20	1.07	−	1.35	0.002

### Comparison of children vs. adults

The RR of difficult-to-transfer cases for adult patients older than or equal to 15 years in 2020 was significant with reference to 2018 (1.25, 95% CI 1.22 to 1.28) (Table 3). With reference to 2018, 2020 was significant for difficult-to-transfer cases for all patients (RR 1.24, 95% CI 1.21 to 1.27) in Multivariate Poisson regression analysis (Table 4). In Table 4, with reference to Friday, all day, Saturday, and Sunday especially had significantly higher RRs for difficult-to-transfer cases (RR 1.26, 95% CI 1.21 to 1.30 and RR 1.38, 95% CI 1.34 to 1.43, respectively). In Table 4, with reference to 9 to 10 am, other times of day were significantly associated with difficult-to-transfer cases. With reference to June, other months were also significantly associated with difficult-to-transfer cases. There was a significant association of patients suspected to have COVID-19 with difficult-to-transfer cases (RR 2.50, 95% CI 2.27 to 2.75).

The severity of illness was shown in Supplemental Table 5 and Table 6. In Supplemental Table 5, the proportion of difficult-to-transfer cases of children in severe and moderate patients was comparable between pre-pandemic years and 2020. In Supplemental Table 6, the proportion of difficult-to-transfer cases of all patients in serious, severe, and moderate patients was higher in 2020 than pre-pandemic years.

## Discussion

Children were associated with a reduced RR for difficulty in obtaining hospital acceptance for transfer of a patient (difficult-to-transfer cases) (RR 0.35, 95% CI 0.33 to 0.37, *P* < 0.001) (Table 4). In contrast, adult patients had a greater RR for difficult-to-transfer cases than the other population (RR 2.87, 95% CI 2.71 to 3.04) (Supplemental Table 1).

During the 3-year study period, 1,147 children were difficult-to-transfer cases (397, 433, and 317 in 2018, 2019, and 2020, respectively). However, it is important to reduce difficult-to-transfer cases, with or without the COVID-19 pandemic. There was a strong association of difficult-to-transfer cases with patients suspected to have COVID-19 in all age patients, but, interestingly, it was not applicable in children (Table 5). As a previous study showed, hospitals were most likely to accept patients of all ages in the morning (9 am to 10 am), on Fridays, and in the month of June, but this was not applicable to children (Table 5) [11]. In children, the year 2020 was not significantly associated with difficult-to-transfer cases, but the year was significantly associated with hospital admission at the ED (RR 1.14, 95% CI 1.10 to 1.18, *P* < 0.001), even though children had a reduced RR for hospital admission from the ED (RR 0.51, 95% CI 0.50 to 0.51, *P* < 0.001) (Supplemental Table 2 and Supplemental Table 4). The exact reason why children had a greater RR of admission in 2020 than the other two years is unclear. One of the reasons for this finding might be due to withholding the medically unnecessary EMS transports during the COVID-19 pandemic in 2020, as shown in Table 3 and Supplemental Table 2. In fact, COVID-19 was significantly associated with hospital admission from the ED (RR 5.27, 95% CI 4.06 to 6.85, *P* < 0.001). Table 6 shows an all-children analysis to investigate which diseases contributed to difficult-to-transfer cases. Injury and poisoning, genitourinary diseases and musculoskeletal diseases were associated with difficult-to-transfer cases for three years, and injury and poisoning were strongly associated with difficult-to-transfer cases in 2020, which was not thought to be related to COVID-19 (Table 6 and Supplemental Table 3). However, diseases of the musculoskeletal system and connective tissue was inversely　associated with hospital admission from the ED (Supplemental Table 2).

Neoplasms, diseases of the circulatory system, diseases of the nervous system, and congenital diseases had higher RRs of hospital admission than other diseases (Supplemental Table 2). This result was similar to other studies [16–18].

Multisystem inflammatory syndrome in children (MIS-C) is characterized by COVID-19-associated Kawasaki disease-like symptoms together with cardiac inflammation and toxic shock syndrome [19]. Kawasaki disease data were collected as ICD-10 code with M303, and 43 patients (19 in 2018, 15 in 2019, and 9 in 2020) were found. In addition, only 7 children had confirmed COVID-19 with admission, and all of them were discharged within 21 days. Thus, diseases of the circulatory system appeared to be different from MIS-C, and MIS-C was not a problem in Osaka Prefecture in 2020. The risk of MIS-C was lower for Omicron variants, compared to Alpha variants and Delta variants in Norway [20]. The proportion of MIS-C cases with severe organ system involvement decreased during widespread Delta and early Omicron circulation compared with before Delta circulation in the US [21]. Duration of hospitalization was shorter and the proportion of pediatric patients who received ICU-level care was significantly lower during widespread Delta and early Omicron circulation [21]. These differences in severity were due to variations in the host immune response, earlier clinical diagnosis, and treatment of MIS-C [21]. There was also a potentially altered clinical phenotype associated with some degree of preexisting immunity conferred by COVID-19 vaccination [21].

Neoplasms, congenital malformations, deformations, and chromosomal abnormalities also contributed to hospital admission from the ED. The caregivers of these patients might delay seeking medical services due to fear of COVID-19 exposure, which resulted in a hospital stay for treatment because most of these patients were immunocompromised [22, 23].

The present results suggest that injury and poisoning were the most common reasons for EMS calls in children and resulted in difficult-to-transfer cases, especially in 2020, but they did contribute to hospital admission from the ED. Pediatric trauma patients may require intensive assessment and care from a multidisciplinary approach [24]. In addition, children often cannot appropriately express their symptoms and signs to parents or EMS personnel, which might be one of the factors associated with the difficulty in hospital acceptance [24]. It is noteworthy that pediatric acute care centers have not been adequately established in Japan compared with other developed countries.

Female sex was associated with difficult-to-transfer cases, however we could not know exact reason why female sex was significantly associated with difficult-to-transfer cases. Girls are more prone to developing urinary tract infection in all age group except first year of life [25]. Connective tissue disorders are also more common in female [26]. Both diseases of the genitourinary system and diseases of the musculoskeletal system and connective tissue were associated with difficult-to-transfer cases more than injury and poisoning. Therefore, we postulate that these diseases might be associated with difficult-to-transfer cases.

There are several limitations in this study. First, COVID-19 is a new disease identified in 2020 in Japan, and acute upper respiratory tract infection or gastroenteritis as ICD-10 code U07.2 in which COVID-19 was suspected was included. Second, one hundred twenty-four patients were left without transportation. ORION system should have recorded the reason why they were not transported, but we could not find the reason. Some of them might be infected with COVID-19, however there were only 2 pediatric patients who were left without transportation. Third, this was a retrospective, observational study, and there might be some unknown confounding factors due to this study type. Fourth, this study uniformly defined the difficult-to-transfer cases regardless of the patient’s condition to assess differences by demographic factors or reasons for ambulance calls. Finally, adjustment for various factors such as medical history, medications, and health status could not be performed in the Poisson regression analysis, because this information was not available.

## Conclusion

The present study found that children had a reduced RR for difficult-to-transfer cases, even in 2020 with the COVID-19 outbreak. Children also had a reduced RR for hospital admission from the ED, but it increased in 2020. Injury and poisoning were the most common reasons for EMS calls and difficult-to-transfer cases.

## Supplementary Information

**Additional file 1: Supplemental Table 1. **Multivariate Poissonregression analysis of difficult-to-transfer cases in all patients (adult).** SupplementalTable 2. **Sensitivity analysis, multivariate Poisson regression analysis of EDdisposition in children only.** Supplemental Table 3. **Sensitivity analysis,multivariate Poisson regression analysis of difficult-to-transfer cases inchildren only in 2020.** Supplemental Table 4. **Sensitivity analysis, multivariatePoisson regression analysis of ED disposition in all patients (children as avariable).** Supplemental Table 5. **Theseverity of illness in children only.** Supplemental Table 6. **The severity of illness inall patients

## Data Availability

The datasets used in the current study are available from the corresponding author on reasonable request.
