# Supplementary material for: Effect of the COVID-19 outbreak on emergency transport of children by an emergency medical service system: a population-based, ORION registry study
Source: BMC Emerg Med. 2022 Dec 20;22:206. doi: 10.1186/s12873-022-00765-8 (PMC9767795; doi:10.1186/s12873-022-00765-8)
Supplement: Supplementary file 1 — Additional file 1: Supplemental Table 1. Multivariate Poissonregression analysis of difficult-to-transfer cases in all patients (adult). SupplementalTable 2. Sensitivity analysis, multivariate Poisson regression analysis of EDdisposition in children only. Supplemental Table 3. Sensitivity analysis,multivariate Poisson regression analysis of difficult-to-transfer cases inchildren only in 2020. Supplemental Table 4. Sensitivity analysis, multivariatePoisson regression analysis of ED disposition in all patients (children as avariable). Supplemental Table 5. Theseverity of illness in children only. Supplemental Table 6. The severity of illness inall patients [file 12873_2022_765_MOESM1_ESM.docx]

Supplemental Table 1. Multivariate Poisson regression analysis of difficult-to-transfer cases in all patients (adult)

|  | Risk Ratio | 95% confidence interval | | | P value |
| --- | --- | --- | --- | --- | --- |
| **Year** |  |  |  |  |  |
| 2018 | Reference |  |  |  |  |
| 2019 | 0.93 | 0.90 | − | 0.95 | <0.001 |
| 2020 | 1.24 | 1.21 | − | 1.27 | <0.001 |
| **Month** |  |  |  |  |  |
| June | Reference |  |  |  |  |
| January | 1.93 | 1.84 | − | 2.02 | <0.001 |
| February | 1.85 | 1.76 | − | 1.94 | <0.001 |
| March | 1.49 | 1.41 | − | 1.57 | <0.001 |
| April | 1.59 | 1.51 | − | 1.67 | <0.001 |
| May | 1.39 | 1.32 | − | 1.47 | <0.001 |
| July | 1.14 | 1.08 | − | 1.20 | <0.001 |
| August | 1.41 | 1.34 | − | 1.49 | <0.001 |
| September | 1.20 | 1.14 | − | 1.27 | <0.001 |
| October | 1.12 | 1.06 | − | 1.18 | <0.001 |
| November | 1.28 | 1.21 | − | 1.35 | <0.001 |
| December | 1.52 | 1.45 | − | 1.60 | <0.001 |
| **Time of transportation** | |  |  |  |  |
| 9 am to 10 am | Reference |  |  |  |  |
| 0 am to 1 am | 6.44 | 5.91 | − | 7.02 | <0.001 |
| 1 am to 2 am | 7.42 | 6.80 | − | 8.08 | <0.001 |
| 2 am to 3 am | 7.95 | 7.29 | − | 8.67 | <0.001 |
| 3 am to 4 am | 7.66 | 7.02 | − | 8.37 | <0.001 |
| 4 am to 5 am | 7.61 | 6.96 | − | 8.31 | <0.001 |
| 5 am to 6 am | 6.80 | 6.21 | − | 7.44 | <0.001 |
| 6 am to 7 am | 5.49 | 5.01 | − | 6.00 | <0.001 |
| 7 am to 8 am | 3.98 | 3.63 | − | 4.36 | <0.001 |
| 8 am to 9 am | 2.16 | 1.97 | − | 2.38 | <0.001 |
| 10 am to 11 am | 1.25 | 1.13 | − | 1.38 | 0.001 |
| 11 am to 12 pm | 1.58 | 1.44 | − | 1.75 | <0.001 |
| 12 pm to 1 pm | 1.92 | 1.75 | − | 2.11 | <0.001 |
| 1 pm to 2 pm | 2.18 | 1.98 | − | 2.39 | <0.001 |
| 2 pm to 3 pm | 2.21 | 2.01 | − | 2.42 | <0.001 |
| 3 pm to 4 pm | 2.41 | 2.19 | − | 2.64 | <0.001 |
| 4 pm to 5 pm | 2.41 | 2.19 | − | 2.64 | <0.001 |
| 5 pm to 6 pm | 3.08 | 2.82 | − | 3.37 | <0.001 |
| 6 pm to 7 pm | 3.85 | 3.54 | − | 4.20 | <0.001 |
| 7 pm to 8 pm | 4.48 | 4.12 | − | 4.88 | <0.001 |
| 8 pm to 9 pm | 4.81 | 4.42 | − | 5.23 | <0.001 |
| 9 pm to 10 pm | 4.86 | 4.46 | − | 5.29 | <0.001 |
| 10 pm to 11 pm | 5.25 | 4.82 | − | 5.72 | <0.001 |
| 11 pm to 0 am | 5.76 | 5.29 | − | 6.28 | <0.001 |
| **Day of week** |  |  |  |  |  |
| Friday | Reference |  |  |  |  |
| Monday | 1.11 | 1.07 | − | 1.15 | <0.001 |
| Tuesday | 1.08 | 1.04 | − | 1.12 | <0.001 |
| Wednesday | 1.09 | 1.05 | − | 1.13 | <0.001 |
| Thursday | 1.06 | 1.02 | − | 1.10 | 0.004 |
| Saturday | 1.26 | 1.21 | − | 1.30 | <0.001 |
| Sunday | 1.38 | 1.34 | − | 1.43 | <0.001 |
|  |  |  |  |  |  |
| Adult | 2.87 | 2.71 | − | 3.04 | <0.001 |
| Suspected COVID-19 | 2.50 | 2.27 | − | 2.75 | <0.001 |

Supplemental Table 2. Sensitivity analysis, multivariate Poisson regression analysis of ED disposition in children only

| **Admission** | Risk Ratio | 95% confidence interval | | | P value |
| --- | --- | --- | --- | --- | --- |
| Year |  |  |  |  |  |
| 2018 | Reference |  |  |  |  |
| 2019 | 0.99 | 0.96 | − | 1.03 | 0.729 |
| 2020 | 1.14 | 1.10 | − | 1.18 | <0.001 |
|  |  |  |  |  |  |
| Infectious and parasitic diseases | 1.90 | 1.64 | − | 2.20 | <0.001 |
| Neoplasms | 4.02 | 3.24 | − | 4.99 | <0.001 |
| Endocrine, nutritional, and metabolic diseases | 3.18 | 2.67 | − | 3.78 | <0.001 |
| Mental and behavioral disorders | 1.25 | 1.02 | − | 1.53 | 0.034 |
| Diseases of the nervous system | 3.35 | 2.89 | − | 3.88 | <0.001 |
| Diseases of the eye and the ear | 0.56 | 0.37 | − | 0.84 | 0.005 |
| Diseases of the circulatory system | 3.27 | 2.74 | − | 3.90 | <0.001 |
| Diseases of the respiratory system | 1.98 | 1.71 | − | 2.29 | <0.001 |
| Diseases of the digestive system | 1.20 | 1.01 | − | 1.42 | 0.039 |
| Diseases of the skin and subcutaneous tissue | 0.98 | 0.78 | − | 1.24 | 0.885 |
| Diseases of the musculoskeletal system and connective tissue | 0.68 | 0.54 | − | 0.87 | 0.002 |
| Diseases of the genitourinary system | 2.76 | 2.26 | − | 3.37 | <0.001 |
| Congenital diseases | 3.65 | 2.70 | − | 4.93 | <0.001 |
| Symptoms, signs, and abnormal clinical and laboratory findings | 1.95 | 1.69 | − | 2.24 | <0.001 |
| Injury and poisoning | 0.90 | 0.78 | − | 1.04 | 0.142 |
|  |  |  |  |  |  |
| Sex | 0.99 | 0.97 | − | 1.02 | 0.689 |
| Suspected COVID-19 | 5.27 | 4.06 | − | 6.85 | <0.001 |
|  |  |  |  |  |  |
| **Home** | Risk Ratio | 95% confidence interval | | | P value |
| Year |  |  |  |  |  |
| 2018 | Reference |  |  |  |  |
| 2019 | 1.00 | 1.00 | − | 1.01 | 0.615 |
| 2020 | 0.97 | 0.97 | − | 0.98 | <0.001 |
|  |  |  |  |  |  |
| Infectious and parasitic diseases | 0.89 | 0.87 | − | 0.91 | <0.001 |
| Neoplasms | 0.58 | 0.50 | − | 0.68 | <0.001 |
| Endocrine, nutritional, and metabolic diseases | 0.73 | 0.68 | − | 0.77 | <0.001 |
| Mental and behavioral disorders | 0.97 | 0.94 | − | 1.00 | 0.066 |
| Diseases of the nervous system | 0.70 | 0.68 | − | 0.72 | <0.001 |
| Diseases of the eye and the ear | 1.06 | 1.03 | − | 1.10 | <0.001 |
| Diseases of the circulatory system | 0.48 | 0.43 | − | 0.53 | <0.001 |
| Diseases of the respiratory system | 0.88 | 0.86 | − | 0.90 | <0.001 |
| Diseases of the digestive system | 0.97 | 0.95 | − | 0.99 | 0.017 |
| Diseases of the skin and subcutaneous tissue | 1.01 | 0.98 | − | 1.04 | 0.474 |
| Diseases of the musculoskeletal system and connective tissue | 1.05 | 1.02 | − | 1.07 | <0.001 |
| Diseases of the genitourinary system | 0.76 | 0.70 | − | 0.81 | <0.001 |
| Congenital diseases | 0.61 | 0.50 | − | 0.75 | <0.001 |
| Symptoms, signs, and abnormal clinical and laboratory findings | 0.89 | 0.87 | − | 0.90 | <0.001 |
| Injury and poisoning | 1.02 | 1.00 | − | 1.04 | 0.073 |
|  |  |  |  |  |  |
| Sex | 1.00 | 1.00 | − | 1.01 | 0.187 |
| Suspected COVID-19 | 0.41 | 0.27 | − | 0.61 | <0.001 |

Death and transfer could not be analyzed by multivariate Poisson regression analysis due to small numbers.

Supplemental Table 3. Sensitivity analysis, multivariate Poisson regression analysis of difficult-to-transfer cases in children only in 2020.

| **Difficult-to-transfer cases** | Risk Ratio | 95% confidence interval | | | P value |
| --- | --- | --- | --- | --- | --- |
| Injury and poisoning | Reference |  |  |  |  |
| Infectious and parasitic diseases | 0.17 | 0.06 | − | 0.45 | <0.001 |
| Neoplasms | 0.00 | 0.00 | − | 0.00 | <0.001 |
| Endocrine, nutritional, and metabolic diseases | 0.00 | 0.00 | − | 0.00 | <0.001 |
| Mental and behavioral disorders | 0.33 | 0.08 | − | 1.31 | 0.116 |
| Diseases of the nervous system | 0.10 | 0.02 | − | 0.40 | 0.001 |
| Diseases of the eye and the ear | 1.02 | 0.26 | − | 4.02 | 0.983 |
| Diseases of the circulatory system | 0.56 | 0.14 | − | 2.24 | 0.414 |
| Diseases of the respiratory system | 0.12 | 0.05 | − | 0.27 | <0.001 |
| Diseases of the digestive system | 0.32 | 0.13 | − | 0.76 | 0.01 |
| Diseases of the skin and subcutaneous tissue | 0.00 | 0.00 | − | 0.00 | <0.001 |
| Diseases of the musculoskeletal system and connective tissue | 1.27 | 0.68 | − | 2.38 | 0.45 |
| Diseases of the genitourinary system | 1.28 | 0.48 | − | 3.40 | 0.617 |
| Congenital diseases | 0.00 | 0.00 | − | 0.00 | <0.001 |
| Symptoms, signs, and abnormal clinical and laboratory findings | 0.13 | 0.08 | − | 0.21 | <0.001 |
| Male | Reference |  |  |  |  |
| Sex | 1.26 | 1.01 | − | 1.57 | 0.044 |

Supplemental Table 4. Sensitivity analysis, multivariate Poisson regression analysis of ED disposition in all patients (children as a variable)

| **Admission** | Risk Ratio | 95% confidence interval | | | P value |
| --- | --- | --- | --- | --- | --- |
| Year |  |  |  |  |  |
| 2018 | Reference |  |  |  |  |
| 2019 | 1.01 | 1.01 | − | 1.02 | <0.001 |
| 2020 | 1.05 | 1.05 | − | 1.06 | <0.001 |
|  |  |  |  |  |  |
| Infectious and parasitic diseases | 1.17 | 1.07 | − | 1.29 | 0.001 |
| Neoplasms | 2.85 | 2.51 | − | 3.22 | <0.001 |
| Endocrine, nutritional and metabolic diseases | 1.66 | 1.51 | − | 1.82 | <0.001 |
| Mental and behavioral disorders | 0.59 | 0.53 | − | 0.66 | <0.001 |
| Diseases of the nervous system | 1.60 | 1.45 | − | 1.76 | <0.001 |
| Diseases of the eye and the ear | 1.07 | 0.98 | − | 1.18 | 0.143 |
| Diseases of the circulatory system | 2.35 | 2.13 | − | 2.58 | <0.001 |
| Diseases of the respiratory system | 2.44 | 2.22 | − | 2.68 | <0.001 |
| Diseases of the digestive system | 2.29 | 2.08 | − | 2.52 | <0.001 |
| Diseases of the skin and subcutaneous tissue | 1.75 | 1.59 | − | 1.93 | <0.001 |
| Diseases of the musculoskeletal system and connective tissue | 1.25 | 1.14 | − | 1.38 | <0.001 |
| Diseases of the genitourinary system | 1.79 | 1.63 | − | 1.97 | <0.001 |
| Congenital diseases | 1.94 | 1.69 | − | 2.22 | <0.001 |
| Symptoms, signs and abnormal clinical and laboratory findings | 0.72 | 0.66 | − | 0.79 | <0.001 |
| Injury and poisoning | 1.14 | 1.04 | − | 1.25 | 0.007 |
| Male | Reference |  |  |  |  |
| Female | 1.00 | 0.99 | − | 1.00 | 0.26 |
| Suspected COVID-19 | 2.79 | 2.53 | − | 3.07 | <0.001 |
| Children | 0.51 | 0.50 | − | 0.51 | <0.001 |
|  |  |  |  |  |  |
| **Home** | Risk Ratio | 95% confidence interval | | | P value |
| Year |  |  |  |  |  |
| 2018 | Reference |  |  |  |  |
| 2019 | 0.99 | 0.99 | − | 0.99 | <0.001 |
| 2020 | 0.96 | 0.96 | − | 0.96 | <0.001 |
|  |  |  |  |  |  |
| Infectious and parasitic diseases | 0.92 | 0.91 | − | 0.93 | <0.001 |
| Neoplasms | 0.26 | 0.26 | − | 0.27 | <0.001 |
| Endocrine, nutritional and metabolic diseases | 0.75 | 0.74 | − | 0.75 | <0.001 |
| Mental and behavioral disorders | 1.13 | 1.12 | − | 1.14 | <0.001 |
| Diseases of the nervous system | 0.78 | 0.77 | − | 0.79 | <0.001 |
| Diseases of the eye and the ear | 0.97 | 0.96 | − | 0.98 | <0.001 |
| Diseases of the circulatory system | 0.35 | 0.34 | − | 0.35 | <0.001 |
| Diseases of the respiratory system | 0.48 | 0.47 | − | 0.48 | <0.001 |
| Diseases of the digestive system | 0.50 | 0.49 | − | 0.50 | <0.001 |
| Diseases of the skin and subcutaneous tissue | 0.72 | 0.71 | − | 0.74 | <0.001 |
| Diseases of the musculoskeletal system and connective tissue | 0.89 | 0.88 | − | 0.90 | <0.001 |
| Diseases of the genitourinary system | 0.69 | 0.68 | − | 0.70 | <0.001 |
| Congenital diseases | 0.64 | 0.58 | − | 0.71 | <0.001 |
| Symptoms, signs and abnormal clinical and laboratory findings | 1.08 | 1.07 | − | 1.08 | <0.001 |
| Injury and poisoning | 0.93 | 0.92 | − | 0.94 | <0.001 |
| Male | Reference |  |  |  |  |
| Female | 1.01 | 1.00 | − | 1.01 | <0.001 |
| Suspected COVID-19 | 0.28 | 0.26 | − | 0.29 | <0.001 |
| Children | 1.26 | 1.26 | − | 1.27 | <0.001 |

Supplemental Table 5. The severity of illness in children only

| 2018 |  |  |  |  |  |
| --- | --- | --- | --- | --- | --- |
| EMS personnel assessment | | | |  |  |
| Severity | Not difficult-to-transfer cases, % | | Difficult-to-transfer cases, % | | Total |
| High | 3,360 | (99.1) | 32 | (0.9) | 3,392 |
| Middle | 29,522 | (98.8) | 345 | (1.2) | 29,867 |
| Low | 1,899 | (99.0) | 19 | (1.0) | 1,918 |
| Non-emergency | 136 | (99.3) | 1 | (0.7) | 137 |
| Physician assessment | | | | |  |
| Death | 68 | (100.0) | 0 | 0.0 | 68 |
| Severe | 71 | (100.0) | 0 | 0.0 | 71 |
| Moderate | 4,451 | (98.4) | 73 | (1.6) | 4,524 |
| Mild | 30,324 | (98.9) | 324 | (1.1) | 30,648 |
| Others | 3 | (100.0) | 0 | 0.0 | 3 |
| Total | 34,917 | (98.9) | 397 | (1.1) | 35,314 |
| 2019 |  |  |  |  |  |
| EMS personnel assessment | | |  |  |  |
| Severity | Not difficult-to-transfer cases, % | | Difficult-to-transfer cases, % | | Total |
| High | 4,832 | (99.4) | 28 | (0.6) | 4,860 |
| Middle | 23,795 | (98.8) | 298 | (1.2) | 24,093 |
| Low | 5,295 | (98.8) | 65 | (1.2) | 5,360 |
| Non-emergency | 3,192 | (98.7) | 42 | (1.3) | 3,234 |
| Physician assessment | | | |  |  |
| Death | 57 | (100.0) | 0 | 0.0 | 57 |
| Severe | 88 | (100.0) | 0 | 0.0 | 88 |
| Moderate | 5,008 | (98.4) | 81 | (1.6) | 5,089 |
| Mild | 31,957 | (98.9) | 352 | (1.1) | 32,309 |
| Others | 4 | (100.0) | 0 | 0.0 | 4 |
| Total | 37,114 | (98.8) | 433 | (1.2) | 37,547 |
| 2020 |  |  |  |  |  |
| EMS personnel assessment | | |  |  |  |
| Severity | Not difficult-to-transfer cases, % | | Difficult-to-transfer cases, % | | Total |
| High | 3,112 | (99.3) | 21 | (0.7) | 3,133 |
| Middle | 16,600 | (98.6) | 229 | (1.4) | 16,829 |
| Low | 2,530 | (98.8) | 32 | (1.2) | 2,562 |
| Non-emergency | 2,138 | (98.4) | 35 | (1.6) | 2,173 |
| Physician assessment | | | |  |  |
| Death | 48 | (100.0) | 0 | 0.0 | 48 |
| Severe | 65 | (100.0) | 0 | 0.0 | 65 |
| Moderate | 3,412 | (98.3) | 58 | (1.7) | 3,470 |
| Mild | 20,853 | (98.8) | 259 | (1.2) | 21,112 |
| Others | 2 | (100.0) | 0 | 0.0 | 2 |
| Total | 24,380 | (98.7) | 317 | (1.3) | 24,697 |

Supplemental Table 6. The severity of illness in all patients

| 2018 |  |  |  |  |  |
| --- | --- | --- | --- | --- | --- |
| EMS personnel assessment | | | |  |  |
| Severity | Not difficult-to-transfer cases, % | | Difficult-to-transfer cases, % | | Total |
| High | 35,471 | (97.4) | 930 | (2.6) | 36,401 |
| Middle | 391,429 | (97.0) | 12,285 | (3.0) | 403,714 |
| Low | 20,291 | (97.7) | 480 | (2.3) | 20,771 |
| Non-emergency | 1,837 | (97.4) | 50 | (2.6) | 1,887 |
| Physician assessment | | | | |  |
| Death | 5,854 | (99.2) | 50 | (0.8) | 5,904 |
| Serious | 15 | (100.0) | 0 | 0.0 | 15 |
| Severe | 8,245 | (98.6) | 117 | (1.4) | 8,362 |
| Moderate | 143,025 | (96.2) | 5,583 | (3.8) | 148,608 |
| Mild | 291,850 | (97.3) | 7,992 | (2.7) | 299,842 |
| Others | 26 | (89.7) | 3 | (10.3) | 29 |
| Total | 449,015 | (97.0) | 13,745 | (3.0) | 462,760 |
| 2019 |  |  |  |  |  |
| Severity | Not difficult-to-transfer cases, % | | Difficult-to-transfer cases, % | | Total |
| High | 45,329 | (97.5) | 1,163 | (2.5) | 46,492 |
| Middle | 321,178 | (97.3) | 9,075 | (2.7) | 330,253 |
| Low | 58,033 | (96.7) | 1,979 | (3.3) | 60,012 |
| Non-emergency | 31,328 | (98.1) | 612 | (1.9) | 31,940 |
| Physician assessment | | | | |  |
| Death | 5,900 | (99.2) | 46 | (0.8) | 5,946 |
| Serious | 8 | (100.0) | 0 | 0.0 | 8 |
| Severe | 8,649 | (98.9) | 95 | (1.1) | 8,744 |
| Moderate | 149,463 | (96.6) | 5,330 | (3.4) | 154,793 |
| Mild | 291,810 | (97.5) | 7,356 | (2.5) | 299,166 |
| Others | 38 | (95.0) | 2 | (5.0) | 40 |
| Total | 455,868 | (97.3) | 12,829 | (2.7) | 468,697 |
| 2020 |  |  |  |  |  |
| Severity | Not difficult-to-transfer cases, % | | Difficult-to-transfer cases, % | | Total |
| High | 41,479 | (96.0) | 1,718 | (4.0) | 43,197 |
| Middle | 294,257 | (96.3) | 11,391 | (3.7) | 305,648 |
| Low | 38,056 | (95.7) | 1,707 | (4.3) | 39,763 |
| Non-emergency | 25,668 | (97.3) | 711 | (2.7) | 26,379 |
| Physician assessment | | | | |  |
| Death | 6,017 | (99.0) | 59 | (1.0) | 6,076 |
| Serious | 5 | (100.0) | 0 | 0.0 | 5 |
| Severe | 8,438 | (98.3) | 148 | (1.7) | 8,586 |
| Moderate | 139,525 | (95.1) | 7,185 | (4.9) | 146,710 |
| Mild | 245,437 | (96.8) | 8,133 | (3.2) | 253,570 |
| Others | 38 | (95.0) | 2 | (5.0) | 40 |
| Total | 399,460 | (96.3) | 15,527 | (3.7) | 414,987 |
